# Little evidence for switches to environmental sex determination and turnover of sex chromosomes in lacertid lizards

**DOI:** 10.1038/s41598-019-44192-5

**Published:** 2019-05-24

**Authors:** Michail Rovatsos, Jasna Vukić, Agata Mrugała, Grzegorz Suwala, Petros Lymberakis, Lukáš Kratochvíl

**Affiliations:** 1 Department of Ecology, Faculty of Science, Charles University, Viničná 7, 12844 Prague 2, Czech Republic; 2 Institute of Animal Physiology and Genetics, Academy of Science of the Czech Republic, Liběchov, Czech Republic; 3 Natural History Museum of Crete, University of Crete, Knossou Avenue, 71409 Irakleio, Crete Greece

**Keywords:** Cytogenetics, Molecular evolution, Evolutionary genetics

## Abstract

Amniotes possess variability in sex determination, from environmental sex determination (ESD), where no sex chromosomes are present, to genotypic sex determination (GSD) with highly differentiated sex chromosomes. Some evolutionary scenarios postulate high stability of differentiated sex chromosomes and rare transitions from GSD to ESD. However, sex chromosome turnovers and two independent transitions from highly differentiated ZZ/ZW sex chromosomes to ESD were previously reported in the lacertid lizards. Here, we examined the homology of sex chromosomes in the wide phylogenetic spectrum of lacertids and their outgroups by comparing gene copy numbers between sexes in genes previously found to be Z-specific in some lacertids. Our current sampling covers 45 species from 26 genera including lineages supposed to possess a derived sex determining systems. We found that all tested lacertids share homologous differentiated ZZ/ZW sex chromosomes, which were present already in their common ancestor living around 85 million years ago. These differentiated sex chromosomes are not present in amphisbaenians and teiid lizards, the close relatives of lacertids. Our study demonstrates how inaccuracies in data can influence the outcome of phylogenetic reconstructions of evolution of sex determination, in this case they overestimated the number of shifts from GSD to ESD and the rate in turnovers of sex chromosomes.

## Introduction

Sex determination, the process that decides the sex of an individual, is variable among lineages of amniotes^1–3^. Despite the great effort and recent advances, the reconstruction of the ancestral state and transitions between particular sex determination modes in amniotes is still equivocal. Some authors argue that the ancestral state was environmental sex determination (ESD), where sexes do not differ in genotype consistently. According to this scenario, the ancestral ESD is still present in recent crocodiles, the majority of turtles and a few squamate lineages^4^. Furthermore, the transitions from ESD to genotypic sex determination (GSD), where sexes differ in genotypes, should be frequent, but transitions in the opposite direction should be rare. This view might be supported by the shared parts of the molecular machinery of sex determination across several ESD lineages^5^, which can, however, also reflect independent co-option of the same epigenetic, thermally-sensitive process. Notably, other authors suggested that GSD, and not ESD, was the ancestral state in amniotes^6^. This alternative was supported by the finding that the same syntenic blocks play the role of sex chromosomes in several lineages, which was interpreted as evidence for a homology of these sex-determining systems. But again, homoplasy, in this case independent co-options of the same part of genome as sex chromosomes, cannot be excluded. Sex chromosomes likely evolved independently many times in amniotes and the repeated independent co-option of the same blocks might be a result of a multiple random selection from a limited number of syntenic blocks, or a higher tendency of a syntenic block to be co-opted due to its gene content, particularly due to enrichment of genes involved in gonad differentiation^7^. The ancestral GSD hypothesis suggests repeated transitions from GSD to ESD.

The two scenarios differ in the predictions on the stability of GSD with respect to ESD and hence the frequency of GSD to ESD transitions. Several transitions from GSD to ESD expected under the ancestral GSD hypothesis were suggested in some phylogenetic reconstructions of the evolution of sex determination systems^8–12^, but many of them were put into doubt by some authors^1,3,13^. Two such putative transitions were reported in the lacertid lizards based on published data in *Podarcis pityusensis*^14^ and *Eremias multiocellata*^15–17^. In lacertids, differentiated ZZ/ZW sex chromosomes containing genes with orthologs linked to the shorter arm of chicken (*Gallus gallus*; GGA) chromosome 4 (GGA4p), homologous to the ancestral X chromosome of viviparous mammals, and GGA17 were documented in 18 species. However, differences in morphology of sex chromosomes among lacertids led to the hypothesis that the differentiation of their sex chromosomes occurred repeatedly and independently in different taxa within the family^18^. Furthermore, recent cytogenetic evidence from comparative chromosome painting points to the non-homology of sex chromosomes between members of the genera *Iberolacerta* and *Timon* versus *Lacerta schreiberi*, suggesting that there has been a turnover of sex chromosomes within lacertids^19^.

In the current study, we performed a molecular test of homology of sex chromosomes using up to now the densest sampling of lacertids. We aimed to clarify the stability and the age of differentiated sex chromosomes in lacertids and to explore the putative exceptions to the general ZZ/ZW pattern. We included the lineages where derived sex determining system was previously reported, which in the case of the genera *Eremias* and *Podarcis* led to the reconstruction of the transitions from the ancestral GSD to ESD within lacertids^8–10,12^, undermining the ancestral ESD hypothesis for amniotes.

## Material and Methods

### Material collection and DNA isolation

Blood or tissue material were collected from both sexes from 27 species of lacertids and their outgroups, i.e., two species of the legless amphisbaenians of the family Blanidae and three species of the family Teiidae (Table S1). When needed, specimens were temporarily maintained in the Animal Facilities of Faculty of Science, Charles University (Accreditation No. 13060/2014-MZE-17214). All experimental procedures were carried out under the supervision and with the approval of the Ethics Committee of the Faculty of Science, Charles University, followed by the Committee for Animal Welfare of the Ministry of Agriculture of the Czech Republic (Accreditation No. 24773/2008-10001). Genomic DNA was extracted from samples using a DNeasy Blood and Tissue Kit (Qiagen) according to the manufacturer’s protocol. DNA concentration and quality were measured by Nanodrop 2000 Spectrophotometer (Thermo Scientific).

### Test of homology based on quantitative real-time PCR (qPCR)

In ZZ/ZW sex chromosome systems genes linked to the Z chromosome and missing on its degenerated W counterpart differ in gene copy numbers between sexes. In such genes, males (ZZ) have twice as many copies than females (ZW), whereas genes in autosomal or pseudoautosomal regions have equal copy numbers in both sexes. Quantitative Real-Time PCR (qPCR) is a useful tool to estimate a difference in copy number between a male and a female individual of the same species. A relative female-to-male gene dose ratio (r) of 0.5 is expected for the Z-specific genes and 1.0 for the (pseudo)autosomal genes. Thus qPCR analysis can be performed in species across a lineage to test whether the same genes are Z-specific in them as a test of homology and degree of differentiation of sex chromosomes. In previous studies, it was shown that 18 species of lacertids have homologous sex chromosomes and their gene content is homologous to a part of GGA4p and GGA17^20,21^. Here we expand these studies by inclusion of other 27 lacertid and five outgroup species to reliably date the origin of the lacertid sex chromosomes. For qPCR measurement, we used previously designed primers targeting two autosomal genes (*adarb2*, *mecom*) and four putative Z-specific genes in lacertids (*mars2*, *lpar4*, *klhl13*, *angptl2*)^21^. In addition, we designed new primers for one autosomal gene with an ortholog in GGAZ (*smad7*), and four candidate Z-linked genes with orthologs linked to GGA4p (*gab3*, *mbnl3*) and GGA17 (*hspa5*, *lrrc8a*). The gene *mecom* was used as a reference gene for the normalization of the qPCR values. The primer sequences are given in Table S2. For detailed methodology on primer design and qPCR calculations see^22^. The qPCR was performed using a LightCycler II 480 (Roche Diagnostics, Basel, Switzerland) and all samples were run in triplicates. The loci with female-to-male gene dose ratio values in the range 0.25–0.75 were considered Z-specific, and in the range 0.75–1.25 autosomal or pseudoautosomal. We tested significance of deviations of the gene dose ratios in lacertids and outgroups in each gene from the values 0.5 expected for Z-specific genes and 1.0 expected for (pseudo-)autosomal genes by t-test.

## Results

**Figure 1:**
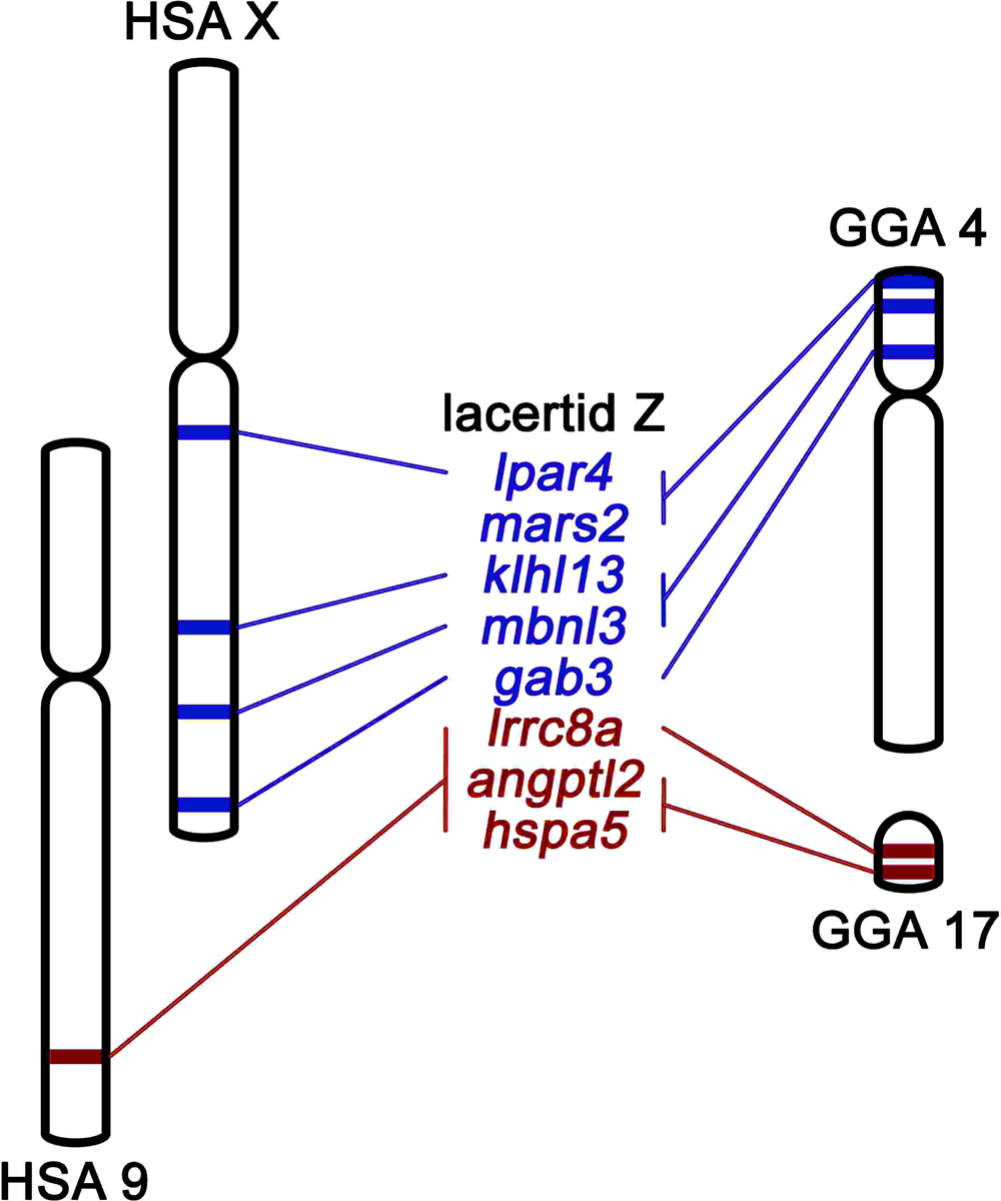
Position of lacertid Z-linked genes in human (HSA) and chicken (GGA) chromosomes.

**Figure 2:**
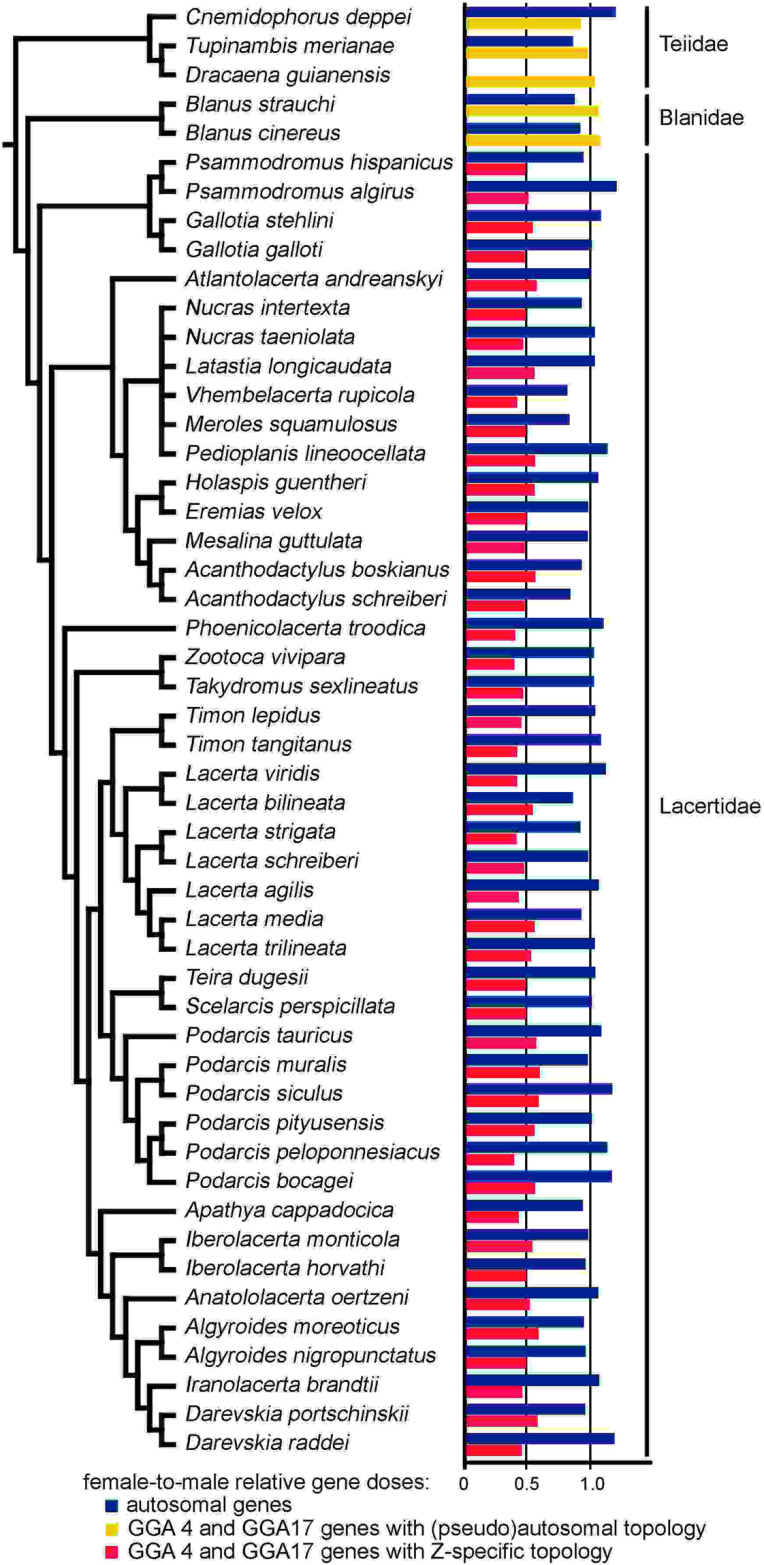
Relative gene dose ratios between sexes in 45 species of lacertids and five species representing outgroups (teiids and amphisbaenians from the family Blanidae). Red and yellow bars correspond to average Z-specific and (pseudo)autosomal values, respectively. Blue bars correspond to the average values for (pseudo)autosomal control loci. Value 1.0 is expected for (pseudo)autosomal genes, while value 0.5 is consistent with Z-specificity. Our results suggest that sex chromosomes are highly conserved and homologous across lacertids, although in some species several genes, which are Z-linked in the majority of lacertids, have a (pseudo)autosomal pattern. These genes were not included in the figure, but were assigned in Table S3. Data from^20,21^ were included. Phylogeny follows^68^. Not all sub-Saharan species studied here were included in this phylogenetic hypothesis, which led to the soft polytomy in this clade.

A relative gene dose was tested by qPCR in four genes previously showed to be Z-linked in lacertids, three of which are orthologous to genes in GGA4p and one to GGA17^20,21^, in 27 species of lacertids and five species of their closest outgroups (two species of blanids and three species of teiids). Two additional genes with orthologs linked to GGA4p and two genes with orthologs linked to GGA17 were also tested in all species using the primers newly designed for this study. Although not all loci amplified successfully in all the tested species, at least two putative Z-specific genes were tested in all species. Overall, all putative Z-specific genes show the expected female-to-male gene dose ratio value of approximately 0.5 across lacertids, although individual genes depart from this pattern in some species (Tables S3 and S4). Therefore, it seems that all tested lacertid species share at least partially the same Z chromosome, with gene content homologous mainly to two major syntenic chromosome regions in chicken (GGA4p, GGA17) and human (HSA9, HSAX) (Fig. 1). The exception is the gene *mars2*, which has orthologs linked to GGA4p and to HSA2^20^. For the comparison of the partial gene content of the Z-specific part of sex chromosomes across major lineages of lacertid lizards and the estimation of the age of sex chromosomes in lacertids, we included the results of the previous study for 18 lacertid species^21^, and thus obtained the densest dataset so far, including altogether 45 lacertid species and five close outgroup species from the families Blanidae and Teiidae. We found that differentiated sex chromosomes are shared across all 45 species of lacertids, including both lacertid subfamilies (Gallotinae and Lacertinae), and broadly covering the phylogenetic diversity of the group (see Fig. 2). We showed that *Podarcis pityusensis*, previously reported to possess ESD^14^ and thus supposed to lack sex chromosomes, shares the same ZZ/ZW sex chromosomes with other lacertids. Also *Lacerta schreiberi*, previously thought to possess different sex chromosomes^19^, has the same partial gene content of the Z-specific part of sex chromosomes, and thus possess the same sex chromosomes as other lacertids. Although we strongly supported the stability of the differentiated sex chromosomes across the lacertids, some Z-linked genes, both those with orthologs linked to GGA4p and those to GGA17, return a (pseudo)autosomal pattern in a few cases in different species (Table S3). Our results showed that the Z-specific genes of lacertids are (pseudo)autosomal in their closest outgroups, blanids and teiids (Tables S3 and S4). This allows the estimation of the age of lacertid differentiated sex chromosomes, placing their origin between the split between lacertids and amphisbaenians approximately 150 MYA and the basal split of lacertids, i.e., the split between Gallotinae and Lacertinae, approximately 85 MYA (the dating of these events follows ref.^23^).

## Discussion

As far as we know, differentiated sex chromosomes (ZZ/ZW or derived multiple neo-sex chromosomes) were uncovered by previous cytogenetic work in 21 species present in our sample, the qPCR results are in agreement with these cytogenetic observations and moreover suggest that these cytogenetically detectable sex chromosomes are homologous. The qPCR results presented here and in our previous studies^20,21^ represent the first evidence for female heterogamety in further 24 species of lacertids. Furthermore, female heterogamety was uncovered up to now solely by cytogenetics in 27 other lacertid species (reviewed in Table 1). Therefore, evidence for female heterogamety exists in *c*. 20% of the recently recognized species of lacertids. The current analysis supports the long-term stability of differentiated ZZ/ZW sex chromosomes across the whole family Lacertidae (Fig. 2). Lacertid sex chromosomes can be dated back to the Mesozoic epoch and are of comparable age to avian sex chromosomes^24^. The former studies documenting the variability in sex chromosomes in lacertids^18,25^ were based on cytogenetic descriptions without any molecular or cytogenetic marker for testing of homology of sex chromosomes. Differentiated W chromosomes are highly variable in sequence content and heterochromatin distribution^26–28^, which can explain the differences in morphology of lacertid sex chromosomes. Interestingly, we found that the Z chromosome of *Lacerta schreiberi* is homologous to Z of other lacertids, although it was previously reported that the flow-sorted probe containing the Z of *Iberolacerta monticola* hybridized to even number of chromosomes in metaphases from both sexes of *L*. *schreiberi*, which indicated that the ancestor of this species had a turnover of sex chromosomes^19^.

**Table 1:** Overview of lacertid lizards with cytogenetic and qPCR^20,21^,this study evidence for female heterogamety.

Species	Cytogenetic evidence	qPCR evidence
*Algyroides moreoticus*	yes^18^	yes
*Algyroides nigropunctatus*	yes^18^	yes
*Atlantolacerta andreanskyi*	yes^46^	yes
*Darevskia portschinskii*	yes^47^	yes
*Darevskia raddei*	yes^47^	yes
*Eremias velox*	yes^48^	yes
*Gallotia galloti*	yes^49^	yes
*Iberolacerta horvathi*	yes^50^	yes
*lberolacerta monticola*	yes^51^	yes
*Lacerta agilis*	yes^52^	yes
*Lacerta bilineata*	yes^49^	yes
*Lacerta schreiberi*	yes^19^	yes
*Lacerta strigata*	yes^48^	yes
*Lacerta trilineata*	yes^53^	yes
*Lacerta viridis*	yes^51^	yes
*Podarcis siculus*	yes^51^	yes
*Podarcis tauricus*	yes^54^	yes
*Psammodromus algirus*	yes^51^	yes
*Takydromus sexlineatus*	yes^55^	yes
*Teira dugesii*	yes^25^	yes
*Timon lepidus*	yes^25^	yes
*Zootoca vivipara*	yes^56^	yes
*Acanthodactylus boskianus*	no	yes
*Acanthodactylus schreiberi*	no	yes
*Anatololacerta oertzeni*	no	yes
*Apathya cappadocica*	no	yes
*Gallotia stehlini*	no	yes
*Holaspis guentheri*	no	yes
*Iranolacerta brandtii*	no	yes
*Lacerta media*	no	yes
*Latastia longicaudata*	no	yes
*Meroles squamulosus*	no	yes
*Mesalina guttulata*	no	yes
*Nucras intertexta*	no	yes
*Nucras taeniolata*	no	yes
*Pedioplanis lineoocellata*	no	yes
*Phoenicolacerta troodica*	no	yes
*Podarcis bocagei*	no	yes
*Podarcis muralis*	no	yes
*Podarcis peloponnesiaca*	no	yes
*Podarcis pityusensis*	no	yes
*Psammodromus hispanicus*	no	yes
*Scelarcis perspicillata*	no	yes
*Timon tangitanus*	no	yes
*Teira dugesii*	no	yes
*Vhembelacerta rupicola*	no	yes
*Acanthodactylus erythrurus*	yes^25^	no
*Acanthodactylus lineomaculatus*	yes^39^	no
*Darevskia armeniaca*	yes^57^	no
*Darevskia dahli*	yes^58^	no
*Darevskia mixta*	yes^59^	no
*Darevskia rostombekovi*	yes^47^	no
*Darevskia unisexualis*	yes^47^	no
*Darevskia valentini*	yes^59^	no
*Dinarolacerta mosorensis*	yes^60^	no
*Eremias arguta*	yes^61^	no
*Eremias grammica*	yes^62^	no
*Heliobolus lugubris*	yes^63^	no
*Hellenolacerta graeca*	yes^18^	no
*Iberolacerta aranica*	yes^64^	no
*Iberolacerta aurelioi*	yes^64^	no
*Iberolacerta bonnali*	yes^64^	no
*Iberolacerta cyreni*	yes^64^	no
*Iberolacerta galani*	yes^65^	no
*Meroles cuneirostris*	yes^25^	no
*Mesalina olivieri*	yes^52^	no
*Omanosaura jayakari*	yes^66^	no
*Ophisops elegans*	yes^67^	no
*Phoenicolacerta kulzeri*	yes^69^	no
*Phoenicolacerta laevis*	yes^69^	no
*Podarcis melisellensis*	yes^52^	no
*Podarcis hispanica*	yes^18^	no
*Podarcis wagleriana*	yes^25^	no

The loci originally revealed to be Z-specific in *Takydromus sexlineatus*, the first lacertid with known partial gene content of sex chromosomes^20^, are Z-specific in other lacertids as well, but several exceptions exist. In some species, putative Z-specific genes gave (pseudo)autosomal pattern in qPCR (Table S3). The distribution of these values with (pseudo)autosomal pattern does not seem to have any clear phylogenetic pattern. According to phylogenetic position of their bearers, these genes seem to be ancestrally Z-specific in lacertids. Analogous situation was found in the genomic analysis of the differentiation of Z and W chromosomes across birds, demonstrating that the sex chromosome evolution might be unexpectedly complex^24^. There are several, up to now purely speculative explanations for this variability. The observed (pseudo)autosomal pattern in otherwise Z-specific genes in lacertids might reflect a different rate of independent differentiation of the W-specific regions from the ancestral pseudoautosomal state, as was suggested for birds^24^. However, we cannot exclude independent secondary re-emergence of recombination between particular Z and W regions recreating locally the pseudoautosomal state or independent translocations of the genes to pseudoautosomal region or autosomes. Alternatively, the scattered pseudoautosomal pattern in certain genes can reflect convergence, for instance by gene conversion, of gametolog sequences leading to binding of qPCR primers otherwise specific to Z gametologs to both Z- and W-linked gametologs. These possibilities should be evaluated in future when more data on genomics of sex chromosomes in lacertids are available. As criticized already by Harlow^13^, the evidence that *Podarcis pityusensis* possesses ESD is extremely poor. It is based on a single description of the production of one male to ‘10–15 females’ at a single temperature without validation of sexing of juveniles^14,29^. However, this species is still included as having ESD in the majority of phylogenetic analyses of sex determination^8–10,12^ but see^1,3^. The results of our analysis strongly suggest that this species has the same sex-linked region as all other tested lacertids. The shared sex-linkage demonstrates that *Podarcis pityusensis* does not have any derived sex determination system, but instead relies on the ancestral ZZ/ZW sex chromosomes of the lacertids.

More recently, ESD was reported in another lacertid, the viviparous species *Eremias multiocellata*^15–17,30^. This information was included in the subsequent comparative phylogenetic analysis, which led to a reconstruction of the second transition from GSD to ESD in the family Lacertidae^12^. Highly biased sex ratio related to constant temperatures during gestation was reported in the first experimental study in *E*. *multiocellata*^15^. In the follow-up study, the differences in sex ratios among temperatures were much less pronounced in the same species and equal sex ratios were reported from the females that went through gestation in the field and from moderate gestation temperatures^16^. The norm of reaction with equal sex ratios in non-extreme temperatures itself questions the presence of ESD^31^. Neither of these two studies were able to exclude differential mortality of sexes at certain temperatures (known for example in snakes)^32^ or temperature-induced sex reversals (reported in the skink *Bassiana duperreyi* or dragon lizard *Pogona vitticeps*)^33–35^. Moreover, juveniles were sexed by examination of hemipene size (in^15^ also by histology, but methodological details and data from histological sections were not presented), which was not validated, e.g., it was not tested whether hemipene size is not phenotypically plastic in relation to temperature. But the most important argument against ESD in *E*. *multiocellata* is the finding of highly differentiated ZZ/ZW sex chromosomes in this species by molecular cytogenetics. The highly differentiated W chromosome was found in all females, but not in any male examined^17^ and the sample size was adequate to document clear, statistically significant sex-linkage of the genotype to sex. Due to unavailability of genetic samples, we were not able to include *E*. *multiocellata* in the recent study, but other congeneric species possess the typical lacertid well-differentiated sex chromosomes^36^ and homologous sex chromosomes between *E*. *velox* and other lacertids was demonstrated^21^ based on our Z-specific molecular markers.

The diversity in sex determination is unequally distributed among amniotes. Traditionally, it was assumed that unlike birds and mammals, reptiles, i.e., the paraphyletic group of non-avian sauropsids, exhibit rapid and frequent transitions in sex-determining systems^37^. Here, we document the gross stability in homology of sex chromosomes in lacertids since the Mesozoic era. Their subsequent evolutionary change can be documented by three reconstructed origins of multiple sex chromosomes in this lineage^38^, here shown variation in the (pseudo)autosomal versus Z-specific pattern revealed for some genes, and highly dynamic nature of repetitive elements on lacertid W chromosome^19,36,39^. Nevertheless, the previously suggested large variability in sex determination and sex chromosomes in lacertids seems to be inaccurate. Furthermore, it is important to keep in mind that earlier data for some reptile lineages might be questionable. In some cases, sex chromosomes were misidentified and confused with autosomes^40,41^ and in other species the earlier reports on the presence of ESD later appeared to be unreliable. Recently, cytogenetic or molecular evidence for sex chromosomes and (hence GSD) was found for previously assumed “ESD” chameleons^42,43^, varanids^44^, skinks^45^ and lacertids^16^, this study. The inclusion of such erroneous “ESD” species in previous phylogenetic comparative studies^8–10,12^, caused an overestimation of the number of GSD to ESD transitions among amniotes, and undermined the long-term stability of GSD systems. We stress that phylogenetic comparative analyses are sensitive to errors in character states and to make them robust, we have to not only fill in the gaps in species with no data, but also to check and critically evaluate the original data.

## Supplementary information

Tables S1, S2, S4

Table S3

## Data Availability

All data are available in the Supplementary Material.
