# Supplementary material for: Little evidence for switches to environmental sex determination and turnover of sex chromosomes in lacertid lizards
Source: Sci Rep. 2019 May 24;9:7832. doi: 10.1038/s41598-019-44192-5 (PMC6534595; doi:10.1038/s41598-019-44192-5)
Supplement: Supplementary file 1 — Tables S1, S2, S4 [file 41598_2019_44192_MOESM1_ESM.pdf]

Supplementary information to:

Little evidence for switches to environmental sex determination and turnover of sex chromosomes in lacertid lizards

Michail Rovatsos, Jasna Vukić, Agata Mrugała, Grzegorz Suwala, Petros Lymberakis, Lukáš Kratochvíl

Table S1. List of samples of lacertids and their outgroups used in the study.

Family	Subfamily	Species	Sex ♂	Sex ♀
Teiidae	Teiinae	*Cnemidophorus deppei*	1	1
Teiidae	Tupinambinae	*Tupinambis merianae*	1	1
Teiidae	Tupinambinae	*Dracaena guianensis*	1	1
Blanidae	Blaninae	*Blanus strauchi*	1	1
Blanidae	Blaninae	*Blanus cinereus*	1	1
Lacertidae	Gallotiinae	*Psammodromus hispanicus*	1	1
Lacertidae	Gallotiinae	*Psammodromus algirus*	1	1
Lacertidae	Gallotiinae	*Gallotia stehlini*	1	1
Lacertidae	Lacertinae	*Atlantolacerta andreanskyi*	1	1
Lacertidae	Lacertinae	*Nucras intertexta*	1	1
Lacertidae	Lacertinae	*Nucras taeniolata*	1	1
Lacertidae	Lacertinae	*Vhembelacerta rupicola*	1	1
Lacertidae	Lacertinae	*Meroles squamulosus*	1	1
Lacertidae	Lacertinae	*Pedioplanis lineoocellata*	1	1
Lacertidae	Lacertinae	*Mesalina guttulata*	1	1
Lacertidae	Lacertinae	*Acanthodactylus schreiberi*	1	1
Lacertidae	Lacertinae	*Phoenicolacerta troodica*	1	1
Lacertidae	Lacertinae	*Timon lepidus*	1	1
Lacertidae	Lacertinae	*Lacerta schreiberi*	1	1
Lacertidae	Lacertinae	*Lacerta trilineata*	1	1
Lacertidae	Lacertinae	*Teira dugesii*	1	1
Lacertidae	Lacertinae	*Scelarcis perspicillata*	1	1
Lacertidae	Lacertinae	*Podarcis pityusensis*	1	1
Lacertidae	Lacertinae	*Podarcis bocagei*	1	1
Lacertidae	Lacertinae	*Apathya cappadocica*	1	1
Lacertidae	Lacertinae	*Iberolacerta monticola*	1	1
Lacertidae	Lacertinae	*Iberolacerta horvathi*	1	1
Lacertidae	Lacertinae	*Anatololacerta oertzeni*	1	1
Lacertidae	Lacertinae	*Algyroides nigropunctatus*	1	1
Lacertidae	Lacertinae	*Iranolacerta brandtii*	1	1
Lacertidae	Lacertinae	*Darevskia portschinskii*	1	1
Lacertidae	Lacertinae	*Darevskia raddei*	1	1

Table S2: Primers used for the measurement of relative gene dosage by qPCR.

Primer name	Forward primer	Reverse primer	Amplicon size
mecom_1	AGAGACTTGAGACCGTTACCTTTG	CACTGATGGTGTTGCTGGTTTT	162
adarb2_1	CTGCTGGGAATGCGACTGG	GCCTTTCGGAGACTGTGGAG	175
smad7_27	TGTGAAAGGCTGGGGTCAGT	AAAGCAAGCACTCAGAAGGAGA	166
mars2_1	TCTTCTGCCCTTGAACCGTC	GGATGCCCTTGTGAACTACCT	200
lpar4_29	CACCTCTCCCCCATTTGTGATG	TGCTGCTTTGACCCTTTCGT	180
klhl13_47	CTGACCACGCAACATAGCA	CAGAAGGCTCCAATGACCAC	183
mbnl3_1	GCTGCCTTGATTTTGGCTTG	CGGGGCGAGAACGACT	167
gab3_5	CGATGTCTTTGTTGACTTTGTGG	TGATAATGGAGAGGATGAGGAGAAA	166
angptl2_39	GGCACCCGTCACTTTCTG	AATGGCTGTCGTGGGAGT	166
hspa5_1	CCATCACACGCTGGTCAAAG	GGACTGGACAAAAGGGAGGG	163
lrrc8a_3	GGAGGCTGAAGATGGAGTGTG	TCAACAACGAGGGCACCAAG	120

Table S4: Statistics for the test of the deviations of the gene dose ratios in lacertids and outgroups from the values 0.5 expected for Z-specific genes and 1.0 expected for (pseudo)autosomal genes.

a) lacertids

				Test against 0.5		Test against 1.0	
**Gene**	**Mean**	**Std.Err.**	**N**	**t-value**	**p**	**t-value**	**p**
**adarb2_2**	1.03	0.02	27	22.16	**<0.00001**	1.33	0.20
**smad7_27**	1.03	0.02	22	23.46	**<0.00001**	1.29	0.21
**lpar4_29**	0.55	0.04	19	1.51	0.15	-12.65	**<0.00001**
**klhl13_47**	0.48	0.01	26	-1.86	0.08	-43.46	**<0.00001**
**mars2_1**	0.53	0.03	21	0.83	0.42	-14.77	**<0.00001**
**gab3_5**	0.51	0.02	16	0.57	0.58	-20.08	**<0.00001**
**mbnl3_1**	0.56	0.05	16	1.21	0.25	-9.18	**<0.00001**
**hspa5_1**	0.64	0.07	16	1.95	0.07	-5.09	**<0.00001**
**lrrc8a_11**	0.55	0.06	16	0.82	0.43	-8.12	**<0.00001**
**angptl2_39**	0.54	0.03	12	1.23	0.24	-13.58	**<0.00001**

b) outgroups

				Test against 0.5		Test against 1.0	
**Gene**	**Mean**	**Std.Err.**	**N**	**t-value**	**p**	**t-value**	**p**
**adarb2_2**	0.87	0.07	2	5.62	0.11	-2.08	0.29
**smad7_27**	1.00	0.08	4	6.52	**0.01**	0.00	1.00
**gab3_5**	0.93	0.06	3	7.61	**0.02**	-1.17	0.36
**mbnl3_1**	1.13	0.05	4	12.79	**0.001**	2.60	0.08
**hspa5_1**	0.96	0.03	5	16.37	**<0.00001**	-1.42	0.23
**lrrc8a_11**	1.11	0.04	4	16.43	**<0.0001**	3.02	0.06
